# Differential Phosphorylation of GluN1-MAPKs in Rat Brain Reward Circuits following Long-Term Alcohol Exposure

**DOI:** 10.1371/journal.pone.0054930

**Published:** 2013-01-23

**Authors:** Yongsheng Zhu, Yunpeng Wang, Bin Zhao, Shuguang Wei, Ming Xu, Enqi Liu, Jianghua Lai

**Affiliations:** 1 Department of Forensic Science, School of Medicine, Xi’an Jiaotong University, Key Laboratory of Ministry of Public Health for Forensic Science, Xi’an, China; 2 Key Laboratory of Environment and Genes Related to Diseases, Xi’an Jiaotong University, Ministry of Education, Xi’an, China; 3 Key Laboratory of Fertility Preservation and Maintenance, Ningxia Medical University, Ministry of Education, Yinchuan, China; 4 Department of Anesthesia and Critical Care, The University of Chicago, Chicago, Illinois, United States of America; Nathan Kline Institute for Psychiatric Research and New York School of Medicine, United States of America

## Abstract

The effects of long-term alcohol consumption on the mitogen-activated protein kinases (MAPKs) pathway and N-methyl-D-aspartate-type glutamate receptor 1 (GluN1) subunits in the mesocorticolimbic system remain unclear. In the present study, rats were allowed to consume 6% (v/v) alcohol solution for 28 consecutive days. Locomotor activity and behavioral signs of withdrawal were observed. Phosphorylation and expression of extracellular signal-regulated protein kinase (ERK), c-Jun N-terminal kinase (JNK), p38 protein kinase and GluN1 in the nucleus accumbens, caudate putamen, amygdala, hippocampus and prefrontal cortex of these rats were also measured. Phosphorylation of ERK, but not JNK or p38, was decreased in all five brain regions studied in alcohol-drinking rats. The ratio of phospho/total-GluN1 subunit was reduced in all five brain regions studied. Those results suggest that the long-term alcohol consumption can inhibits GluN1 and ERK phosphorylation, but not JNK or p38 in the mesocorticolimbic system, and these changes may be relevant to alcohol dependence. To differentiate alcohol-induced changes in ERK and GluN1 between acute and chronic alcohol exposure, we have determined levels of phospho-ERK, phospho-GluN1 and total levels of GluN1 after acute alcohol exposure. Our data show that 30 min following a 2.5 g/kg dose of alcohol (administered intragastrically), levels of phospho-ERK are decreased while those of phospho-GluN1 are elevated with no change in total GluN1 levels. At 24 h following the single alcohol dose, levels of phospho-ERK are elevated in several brain regions while there are no differences between controls and alcohol treated animals in phospho-GluN1 or total GluN1. Those results suggest that alcohol may differentially regulate GluN1 function and ERK activation depending on alcohol dose and exposure time in the central nervous system.

## Introduction

Alcohol dependence is a complex neuropsychiatric disorder characterized by chronic drinking, abstinence, relapse and behavioral impairments. Long-term consumption of alcohol has been reported to modify a multitude of molecular events such as the function of neurotransmitter receptors, intracellular signal transduction systems and biochemical processes in the central nervous system [1].

Drugs of abuse enhance the activity of the dopaminergic mesocorticolimbic pathway, which arises from the ventral tegmental area (VTA) and projects to the nucleus accumbens (NAc), caudate putamen (CPu), amygdala (Amy), hippocampus (Hip) and the prefrontal cortex (PFC) [2], [3]. The NAc modulates motivation for drug seeking by integrating information from the basolateral Amy and PFC [4]. The CPu is thought to provide a link between motivation and motor outcomes, due to connections between the NAc and CPu via the ventral midbrain [5]–[7]. The Amy projects heavily to the NAc and is involved in conditioned learning of drug reinforcement and drug-associated cues [2]. The Hip plays important roles in the consolidation of information from short-term memory to long-term memory that is thought to be involved in addiction [8]. The PFC send reciprocal connections to the VTA, modulating the activity of this nucleus and its subsequent output to limbic structures [9], while convergent inputs from the Amy, Hip and PFC to the NAc modulates outputs to motor relay circuits that oversee motor actions and outcomes [6], [10], [11]. These pathways coordinate reward-related associative learning and motivated behaviors and limbic-associated pathways by drugs of abuse are thought to contribute the alterations in learning, memory and behavior that underlie addiction [12], [13].

The mitogen-activated protein kinases (MAPKs) pathway represents a converging point for many signaling pathways and can be activated by chronic drug treatments [14]. MAPKs include the extracellular signal-regulated kinase (ERK), the c-Jun N-terminal kinase (JNK) and the p38 protein kinase [15]. Upon phosphorylation, MAPKs translocate to the nucleus and facilitate gene transcription [16]. A mounting body of research demonstrated that ERK is involved in neural plasticity, learning and memory and drug reinforcement [17], [18]. Enhanced ERK phosphorylation has been found in the PFC, the shell of the NAc, the central and basolateral nucleus of the Amy, the paraventricular nucleus of the hypothalamus, and in the Edinger-Westphal nucleus following acute alcohol administration [19], [20]. However, little is known about the effects of chronic alcohol consumption on the phosphorylation of these MAPKs in mesocorticolimbic areas.

The N-methyl-D-aspartate-type glutamate receptors (NMDARs) are heteromeric complexes that incorporate the NMDAR1 (GluN1), NMDAR2, and NMDAR3 subunits. Without GluN1, NMDAR complexes are not functional [21]. GluN1 subunits are also important determinant of alcohol sensitivity [22]. It was suggested that alcohol bound to the third transmembrane domain (TM3) of the GluN1 subunit [23]. Chronic exposure to alcohol induces a number of adaptive processes in the central nervous system, including an upregulation of NMDARs and inhibition of their function [24]. Phosphorylation of GluN1, especially at Ser897, is known to enhance NMDAR activity [25], [26]. It has been demonstrated that NMDARs can activate the MAPK pathway mainly through a Ca^2+^-dependent signaling pathway. Increasing evidence also shows that glutamate receptor-dependent activation of the MAPK pathway is critical for the development of striatal neuronal plasticity and is an important molecular mechanism for the long-lasting behavioral plasticity [27].

Although the association between drug dependence and dysfunction of mesocorticolimbic systems is well documented, the molecular mechanisms and particularly the effects of long-term alcohol consumption on the phosphorylation of MAPKs and GluN1 subunits have not been elucidated. In the present study, we hypothesized that alcohol would alter the phosphorylation of ERK, JNK, p38 and GluN1 in the mesocorticolimbic system of the rat. To test this hypothesis, rats were exposure to a 6% (v/v) alcohol solution for 28 d and a single dose of alcohol (2.5 g/kg, intragastrically). The phosphorylated MAPKs and GluN1 in the NAc, CPu, Amy, Hip and PFC was examined in rats.

## Materials and Methods

### Animals

Sixty-four male Sprague-Dawley rats (Laboratory Animal Center of Xi’an Jiaotong University, China) that were 16 weeks old and weighed 260–280 g at the beginning of the experiment were habituated for 7 d before the experiments. Rats were individually housed in a temperature- and humidity-controlled room (22±2°C and 60±5%, respectively) under a 12 h light/dark cycle (lights on at 8∶00). Food was available *ad libitum*. All experiments were carried out between 9∶00 and 16∶00. All experiments were approved by the Animal Care and Use Committee of Xi’an Jiaotong University, and the Animal Research: Reporting of *In Vivo* Experiments (ARRIVE) guidelines have been followed. Efforts were made to minimize animal suffering and to reduce the number of animals used.

### Drug and Antibodies

Anhydrous ethanol (Huada Pharmaceutical Factory, Guangdong, China) was dissolved in sterile water at a concentration of 6% (v/v). Rabbit anti-phospho-ERK (Thr202/Tyr204), phospho-JNK (Thr183/Tyr185), phospho-p38 (Thr180/Tyr182), phospho-GluN1 (Ser897), ERK, JNK, p38 and GluN1 polyclonal antibodies were purchased from Cell Signaling Technology (Beverly, MA, USA). Mouse anti-β-actin monoclonal antibody and horseradish peroxidase-conjugated anti-rabbit or anti-mouse secondary antibodies were purchased from Santa Cruz Technology (Santa Cruz, CA, USA).

### Experiment 1

#### Long-term alcohol consumption

Rats were assigned randomly to two groups: a control group (n = 10) and an alcohol group (n = 10), receiving water and alcohol solution *ad libitum*, respectively, as the only liquid source during the experimental period. In order to induce alcohol consumption, an alcohol solution was administered at a concentration from 0.5% v/v to 6% v/v for adaptation in the first 12 d and 6% v/v in the following 28 d, as described by Turchan J. et al. [28]. Locomotor activity was assessed after 7, 14, 21 and 28 d of 6% alcohol treatment. At the end of the 28-d treatment, alcohol solution was replaced with water. Withdrawal syndromes were evaluated at different time points. The amount of alcohol consumed (g of pure alcohol per kg of body weight) was recorded daily. The body weight of the rats was monitored weekly.

To minimize the effects of handling and behavioral assessments, a separate cohort of rats was allowed to drink the 6% alcohol solution or water (n = 10 per group) for 28 d. Those rats neither undergo any behavioral tests nor alcohol withdrawal. The rats were sacrificed by decapitation at the end of the 28-d treatment, brains were quickly removed and stored at −80°C until use.

#### Blood Alcohol Levels (BALs)

Blood samples (40 µl) were taken via tail vein at 8∶00–9∶00 after 7, 14, 21 and 28 d of alcohol drinking. Ten µl of plasma was used to determine blood alcohol concentration using an EnzyChrom™ Ethanol Assay Kit (ECET-100, BioAssay Systems, Hayward, CA, USA).

#### Open field test

According to the method of Erden, *et al*. [29], a black rectangular box was used (100×50×60 cm) for open field test. The box was illuminated with three 30 W fluorescent bulbs placed 2 m above the box. The experiment was carried out in a sound-attenuating room at 9∶00. Rats were placed in the central area of the box and allowed 10 min of exploration. The total distance travelled was analyzed by a computerized video-tracking system (SMART, Panlab SL, Barcelona, Spain). Rearing (lifting both fore limbs off the floor) was counted by an observer blind to the treatment.

#### Evaluation of Ethanol Withdrawal Syndrome (EWS)

At the end of the 28-d exposure to 6% alcohol solution, alcohol was withdrawn from the drinking water at 9∶00. The rats were then observed for 10 min at the 0 (before the removal of alcohol), 2, 6, 24, 48 and 72 h of the withdrawal period. At each observation time, rats were assessed simultaneously for the following behavioral signs: stereotyped behavior, agitation (irritability to touch), tail stiffness, abnormal posture and gait. In the study, grooming, sniffing, head weaving, gnawing, and chewing were observed as major stereotypes behaviors during the alcohol withdrawal. Stereotypic behaviors, abnormal posture and gait, agitation, and tail stiffness were scored using a rating scale as previously described (Table S1) [29]. The first 5 min of the scoring period were excluded from the analyses to allow the subjects sufficient time to habituate to the observation cage. The observation was carried out by an observer blind to the treatment.

### Experiment 2

#### Acute alcohol exposure

For acute alcohol exposure, rats were infused intragastrically with 2.5 g/kg ethanol in sterile water, volume administered was equivalent to 0.015 ml/g of body weight of a 21% ethanol solution (based on previous reported method by Carlos Arias *et al.* [30]). Control animals were treated precisely in the same way, but sterile water was administered.

Blood samples were taken at 0 min (before the administration of alcohol), and at 30 min, 2 h, 6 h and 24 h after alcohol administration. Blood alcohol levels (BALs) were determined. To reduce the number of animals used, animals were decapitated when an acute effect was expected and disappared, i.e. 30 min (n = 6) and 24 h (n = 6). Controls (n = 12) were administered with water and killcd with the alcohol-treated rats. Brains were quickly removed and stored at −80°C until use.

#### Western blotting

The NAc, CPu, Amy, Hip, PFC were dissected on ice using a rat brain atlas (Figure S1). Brain tissues were homogenized in a pre-cooled RIPA buffer (50 mM Tris–HCl pH 7.5, 50 mM NaCl, 5 mM EDTA, 10 mM EGTA, 2 mM sodium pyrophosphate, 4 mM paranitrophenylphosphate, 1 mM sodium orthovanadate, 1 mM phenylmethylsulfonyl ﬂuoride, 2 µg/ml aprotintin, 2 µg/ml leupeptin and 2 µg/ml pepstatin). The homogenates were incubated on ice for 30 min and centrifuged at 12,000×g for 15 min at 4°C. The protein content was determined using a bicinchoninic acid method (Joincare Co., Zhuhai, China). The protein samples were subjected to 12% SDS-PAGE and transferred to PVDF membranes. The membranes were blocked with 5% fat milk in Tris-buffered saline (TBS) (500 mM NaCl, 20 mM Tris-HCl pH 7.5) containing 0.05% Tween-20 for 1 h and incubated overnight with one of the following antibodies at 4°C: primary antibodies against phospho-ERK, phospho-JNK, phospho-p38, phospho-GluN1 at a 1∶1000 dilution. The same dilution was used for the antibodies against total protein. The next day, the membranes were washed three times with 0.1% Tween-20 TBS (pH 7.6) and incubated with horseradish peroxidase-conjugated anti-rabbit or anti-mouse secondary antibodies. An enhanced chemiluminescence kit (Millipore, MA, USA) was used to detect immunoreactive protein bands. The blots were normalized to β-actin (1∶5000).

#### Data analyses

Data analyses were performed using SPSS (Ver 13.0, SPSS Inc., USA). Alcohol consumption and BALs were determined using a one-way analysis of variance (ANOVA) with repeated measures. Weight gain, total fluid intake, locomotor activity, global EWS scores were compared between the two treatment groups using two-way ANOVA with repeated measures. Immunoreactive protein bands were quantified by densitometry using the Quantity One (BioRad, Hercules, US). Proteins levels in the water-drinking control rats were set at 100%. Independent Student’s t-tests were conducted to analyze immunoreactivity changes in MAPKs and GluN1. All data are presented as means ± the standard deviation (SD). Statistical significance was accepted at *p*<0.05.

## Results

### 1. Experiment 1: Long-term Alcohol Exposure

#### 1.1. Weight gain, alcohol consumption and BALs

**Figure 1:**
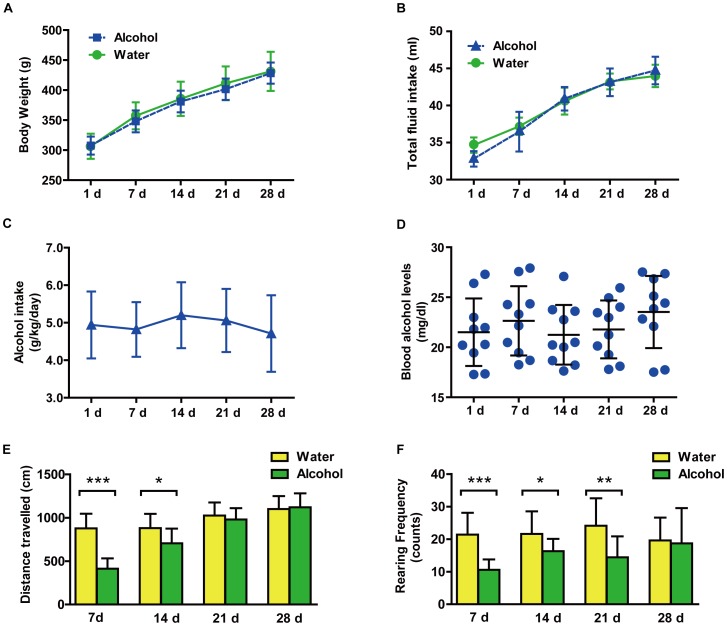
Effects of long-term alcohol drinking on body weight, total fluid intake, alcohol consumption, blood alcohol levels (BALs) and locomotor activity. (A) Changes in the body weight of rats (n = 20 per group) during the 28 d experimental period. (B) Fluid intake (ml) of the rats (n = 20 per group) during the 28 d experimental period. (C) Alcohol consumption (g of pure alcohol per kg of body weight, n = 10) and (D) BALs (mg/dl, n = 10) during a 28 d alcohol-drinking period. (D) The total distance travelled (E) and rearing frequency were also observed (n = 10 per group). The values represent the mean ± SD. *p<0.05; **p<0.01; ***p<0.0001 *vs.* water controls or 0 h.

No significant differences in weight gain between the alcohol- and water-drinking rats were noted [treatment: F_(4,190)_ = 2.54, *p* = 0.1128] (Fig. 1, A). All rats exhibited an increase in body weight during the alcohol- or water-drinking period [time: F_(1,190)_ = 179, *p*<0.0001]. The mean body weight gain for the period was 121 g in the alcohol-drinking group and 125 g in water-drinking control group.

Total fluid intake did not differ between the alcohol- and water-drinking rats [treatment: F_(4,190)_ = 0.11, *p* = 0.7454] (Fig. 1, B). All rats exhibited an increase in fluid intake during the alcohol- or water-drinking period [time: F_(1,190)_ = 14.37, *p*<0.0001]. Rats stably consumed an average of 4.85±0.79 g/kg/day of alcohol in their home cages for 28 d [F_(4,36)_ = 2.45, *p* = 0.64] (Fig. 1, C), which resulted in average BALs of 22.35±1.3 mg/dl. The BALs did not differ significantly over the alcohol-drinking period [F_(4,36)_ = 0.51, *p* = 0.7276] (Fig. 1, D).

#### 1.2. Evaluation of physical dependence

Repeated measures ANOVA revealed a significant treatment–time interaction [F_(3,54)_ = 11.23, *p*<0.0001] in total distance traveled, which increased over the 28 days period in both groups [time effect: F_(3,54)_ = 42.28, *p*<0.0001] (Fig. 1, E). The total distance travelled was dramatically lower in alcohol-drinking rats at 7 and 14 d (post hoc, *p*<0.0001 and *p*<0.05 *vs.* water-control group, respectively). Repeated measures ANOVA analysis of rearing behavior revealed a significant treatment–time interaction [F_(3,54)_ = 5.45, *p* = 0.0232] but no time effect [F_(3,54)_ = 1.17, *p* = 0.3293] (Fig. 1, F). Differences between the two groups were significant at 7, 14 and 21 d (post hoc, *p*<0.0001, *p*<0.05, and *p*<0.01 *vs.* water-control group, respectively).

**Figure 2:**
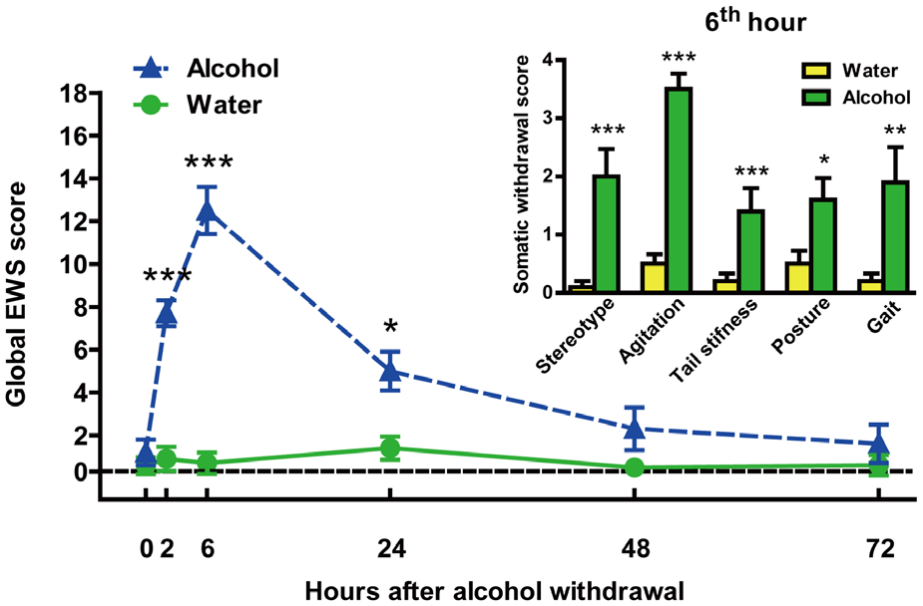
Global withdrawal severity (sum of somatic withdrawal scores across the 5 behavioral signs) measured between 2 and 72 hours after the removal of the 6% v/v alcohol solution. (Inset) Somatic withdrawal signs measured at 6^th^ h of the alcohol withdrawal (n = 10 per group). Rats of water-drinking group were evaluated as a control. The values represent the mean ± SD. *p<0.05; **p<0.01; ***p<0.0001 different from water-control group;

Alcohol-drinking rats showed significant withdrawal signs (compared with water-control rats) at different withdrawal time points [treatment: F_(1,108)_ = 111.8, *p*<0.0001; time: F_(5,108)_ = 20.66, *p*<0.0001] (Fig. 2). Moreover, two-way ANOVA revealed a significant treatment-time interaction [F_(5,108)_ = 19.14, *p*<0.0001] in those rats. The sum of the 5 observation scores (stereotype behaviors, agitation, tail stiffness, abnormal posture and gait) progressively increased from 7.7±0.6 at 2 h (Dunnett’s post hoc test, *p*<0.0001 *vs.* water-control) to 5.1±0.9 at 24 h (*p*<0.05), with a peak score around 6 h (score: 12.5±1.1; *p*<0.05), confirming the presence of significant overall withdrawal severity. All individual withdrawal ratings (compared with water-control rats), measured at 6 h after withdrawal (Fig. 2 inset), were significantly elevated as confirmed by a significant increase in stereotype behaviors (*p*<0.0001 ), agitation (*p*<0.0001), tail stiffness (*p*<0.0001), abnormal posture (*p*<0.05) and gait (*p*<0.01).

#### 1.3. MAPKs and GluN1 phosphorylation in the NAc, CPu, Amy, Hip and PFC

**Figure 3:**
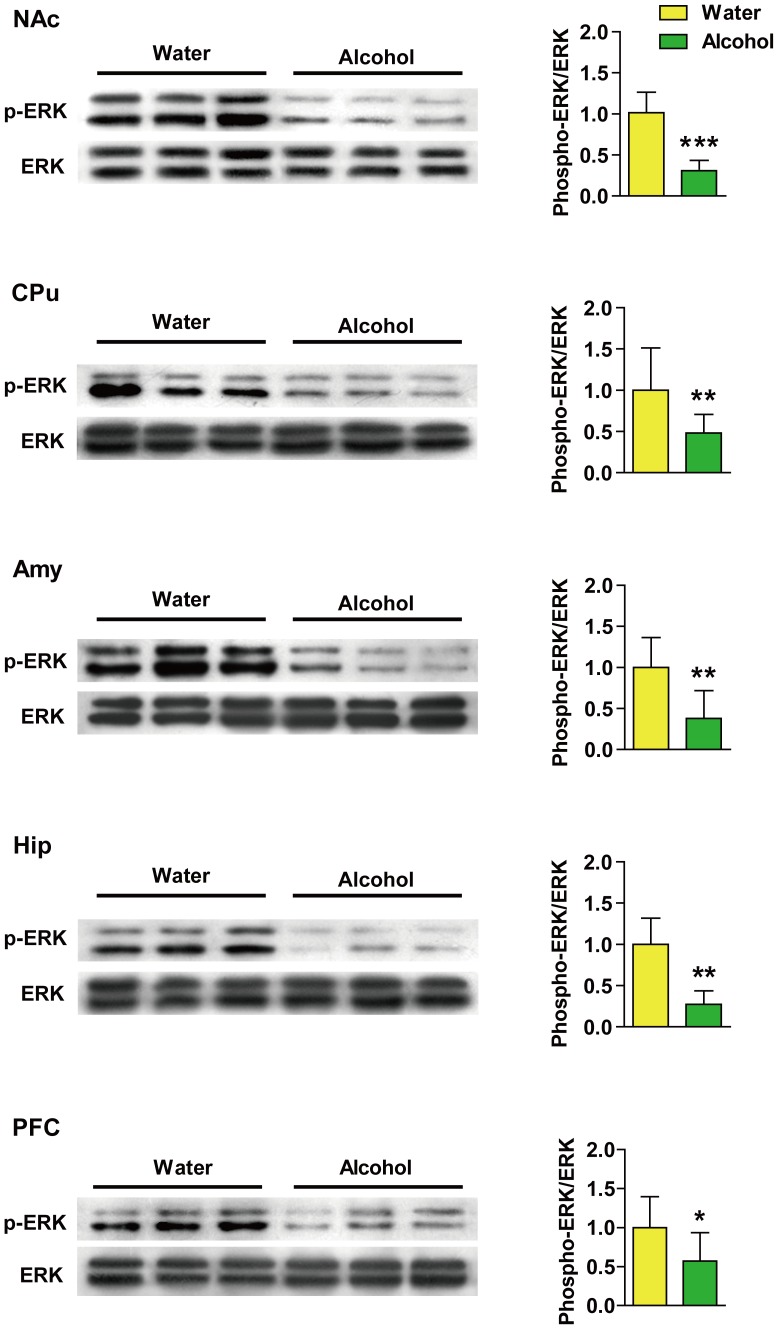
Decreased extracellular signal-regulated kinase (ERK) (Thr202/Tyr204) phosphorylation was found in the nucleus accumbens (NAc), caudate putamen (CPu), amygdala (Amy), hippocampus (Hip) and the prefrontal cortex (PFC) in rats following 28 d of 6% (v/v) alcohol exposure. Ratios of phospho/total-ERK protein levels in the five brain regions were analyzed. Data were expressed as mean ± SD relative to water-drinking controls that were set as 100%. β-actin was used as loading control. *p<0.05, **p<0.01, ***p<0.001 *vs.* water-controls.

**Figure 4:**
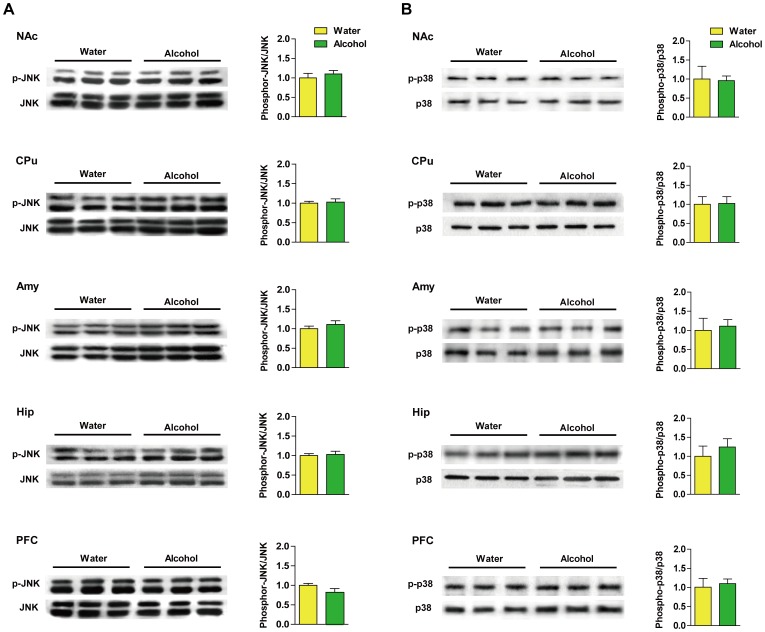
Phosphorylated c-*jun* N-terminal kinase (JNK) (Thr783/Tyr185) and p38 (Thr180/Tyr182) are not obviously changed in alcohol-drinking rats following 28 d of 6% (v/v) alcohol exposure. Ratios of (A) phospho/total-JNK and (B) p38 protein levels in the nucleus accumbens (NAc), caudate putamen (CPu), amygdala (Amy), hippocampus (Hip) and the prefrontal cortex (PFC) were analyzed. Data represent mean ± SD relative to water-drinking controls that were set as 100%. β-actin was used as a loading control.

Following 28 d of 6% (v/v) alcohol drinking, phospho-ERK (Thr202/Tyr204) was attenuated in the NAc (*p*<0.0001), CPu (*p*<0.01), Amy (*p*<0.01), Hip (*p*<0.01) and PFC (*p*<0.05) of rats as compared with the water-control rats (Fig. 3). No obvious change was observed in the phosphorylation of JNK (Thr183/Tyr185) (Fig. 4, A) or p38 (Thr180/Tyr182) (Fig. 4, B) in any of the brain regions compared with the controls. The expression of total-ERK, JNK and p38 was not different between the alcohol-drinking and water-control rats in any of those brain regions (data not shown).

**Figure 5:**
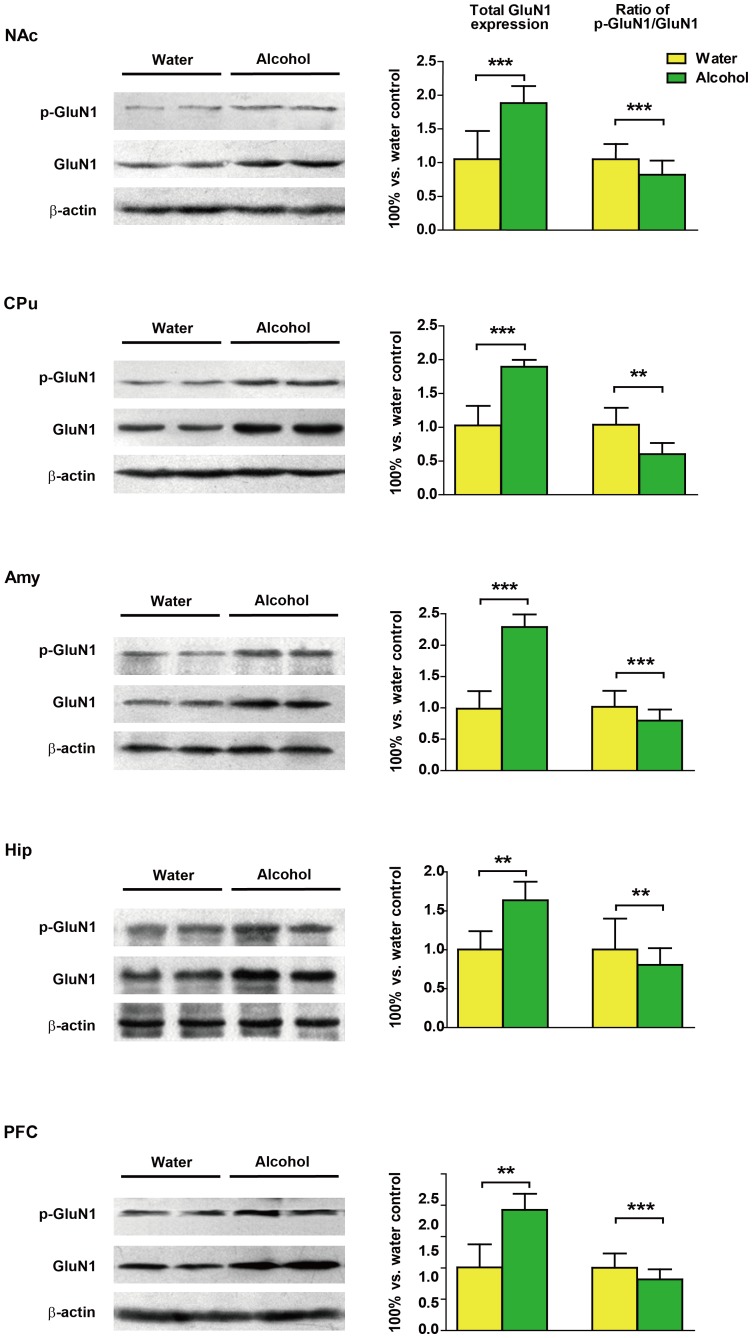
Effects of long-term alcohol intake on N-methyl-D-aspartate-type glutamate receptors 1 (GluN1) expression and phosphorylation (Ser897) in the nucleus accumbens (NAc), caudate putamen (CPu), amygdala (Amy), hippocampus (Hip) and the prefrontal cortex (PFC). Total-GluN1 expression was significantly increased. Obvious decreases were found in the phospho/total-GluN1 in all of the five brain regions examined. Data represent mean ± SD relative to water-drinking controls that were set as 100%. β-actin was used as a loading control. *p<0.05, **p<0.01, ***p<0.001 *vs.* water-controls.

Western blots analysis of total-GluN1 and phospho-GluN1 the NAc (*p*<0.0001), CPu (*p*<0.0001), Amy (*p*<0.0001), Hip (*p*<0.01) and PFC (*p*<0.01) showed significantly higher levels in the alcohol-drinking rats compared to the control rats (Fig. 5). There was also significant decrease of the proportion of phosphorylation of GluN1 (Ser897)/total-GluN1 was found in the alcohol-drinking rats (Fig. 5, NAc: *p*<0.0001, CPu: *p*<0.01, Amy: *p*<0.0001; Hip: *p*<0.01, PFC: *p*<0.0001) *vs.* control rats.

### 2. Experiment 2: Acute Alcohol Exposure

#### 2.1. BALs

**Figure 6:**
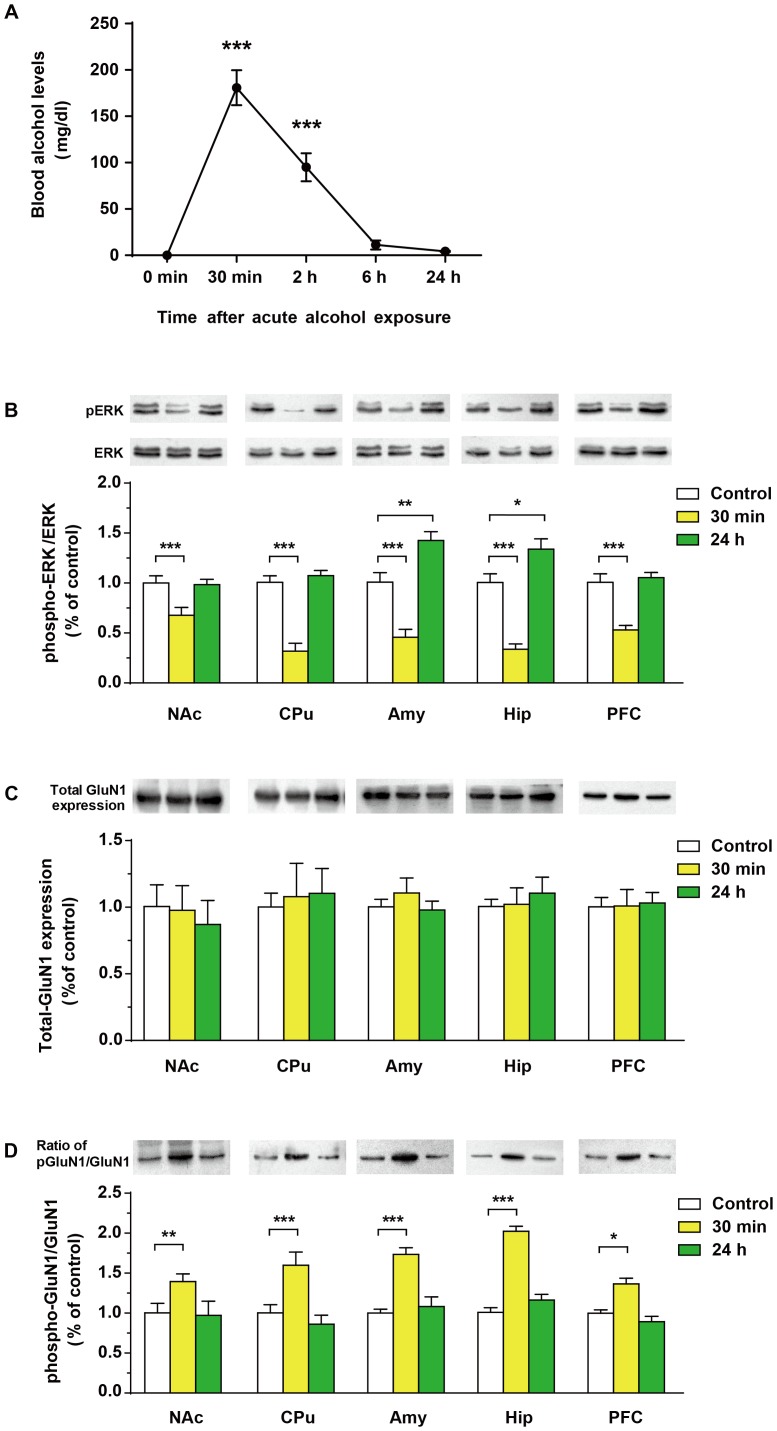
Effects of a single exposure of alcohol on blood alcohol levels (A), and ratios of phospho/total-ERK (B), total GluN1 (C), ratios of phospho/total-GluN1 (D) protein levels in the nucleus accumbens (NAc), caudate putamen (CPu), amygdala (Amy), hippocampus (Hip) and the prefrontal cortex (PFC) in rats following a single dose of alcohol exposure. Data were expressed as mean ± SD. For panel A, ***p<0.0001 *vs.* 0 min (before alcohol administration). For panel B, C and D, water-controls were set as 100%, *p<0.05, **p<0.01, ***p<0.001 *vs.* water-controls.

The BALs differed significantly over the acute alcohol-withdrawal period [F_(4,20)_ = 57, p = 0.0029] (Fig. 6, A). The BAL was 180.83±46 mg/dl at 30 min and 95±16 mg/dl at 2 h. The BAL was barely detectable at 6 (11.3±3.4 mg/dl) and 24 h (4.3±0.65 mg/dl).

#### 2.2. ERK phosphorylation in the NAc, CPu, Amy, Hip and PFC

Thirty minutes after a single dose of alcohol administration, phospho-ERK (Thr202/Tyr204) was significantly attenuated in the NAc (main effect: F_(2,15)_ = 20.72, p<0.0001; post hoc: p<0.0001), CPu (main effect: F_(2,15)_ = 76.6, p<0.0001; post hoc: p<0.0001), Amy (main effect: F_(2,15)_ = 60.0, p<0.0001; post hoc: p<0.0001), Hip (main effect: F_(2,15)_ = 54.2, p<0.0001; post hoc: p<0.0001) and PFC (main effect: F_(2,15)_ = 42.0, p<0.0001; post hoc: p<0.0001) of rats as compared with the water-controls (Fig. 6, B). Tween-four hours later, however, significant elevation of phospho-ERK was observed in Amy (post hoc: p<0.01) and Hip (post hoc: p<0.05). No obvious change was observed in the phospho-ERK in NAc, CPu and PFC when compared with the controls (Fig. 6, B). The expression of total-ERK was not different between the alcohol and water-control rats in any of those brain regions (data not shown).

#### 2.3. GluN1 phosphorylation in the NAc, CPu, Amy, Hip and PFC

Western blots analysis of total-GluN1 expression showed no significant change in the NAc (F_(2,15)_ = 0.98, p = 0.39), CPu (F_(2,15)_ = 0.74, p = 0.63), Amy (F_(2,15)_ = 0.68, p = 0.51), Hip (F_(2,15)_ = 0.26, p = 0.76) and PFC (F_(2,15)_ = 0.02, p = 0.97) in the alcohol rats compared to the controls (Fig. 6, C).

Thirty minutes after a single dose of alcohol administration, significant increase of the proportion of phosphorylation of GluN1(Ser897)/total-GluN1 was found in NAc (main effect: F_(2,15)_ = 18.33, p<0.0001; post hoc: p<0.01), CPu (main effect: F_(2,15)_ = 54.46, p<0.0001; post hoc: p<0.0001), Amy (main effect: F_(2,15)_ = 20.42, p<0.0001; post hoc: p<0.00001), Hip (main effect: F_(2,15)_ = 73.21, p<0.0001; post hoc: p<0. 01), PFC (main effect: F_(2,15)_ = 16.54, p<0.01; post hoc: p<0.05) in the alcohol rats (Fig. 4). Tween-four hours later, no obvious change was observed in the phospho-GluN1/total-GluN1 in all five brain regions (Fig. 6, D).

## Discussion

In our experiments, no significant differences were found in body weight between the alcohol-drinking and control rats, suggesting that the food intake is not affected during the 28 d of 6% alcohol drinking period. Rats stably consumed an average of 4.85±0.79 g/kg/day of alcohol in their home cages for 28 d, which resulted in relatively lower BALs (average: 22.35±1.3 mg/dl). Shah et al. reported that the blood alcohol levels (BALs) in Swiss-Webster mice reached 20.06±8.32 mg/dl on the first day, and 14.75±3.98 mg/dl on the 7th day, by administrating a 7% v/v alcohol solution for 7 d [9]. Furthermore, the report by Barson showed that the BALs in SD rats reached 18.9±4.0 mg/dl by administrating an alcohol solution of increasing concentration from 1% to 7% v/v for 16 d [10]. In our study, the blood samples were taken from the tail vein at 9∶00 AM. The BALs was low because the rats consumed alcohol at a different time points at the dark phase. The rats with lower BAL may consume ethanol in the early period of the dark phase, whereas the rats with higher BAL probably consumed alcohol in the later period of the dark phase [31]. Taken together, those results indicate that the alterations in behavior and protein expression in the alcohol-drinking rats resulted from adaptations in brain function rather than insufficient food intake or alcohol accumulation in the blood.

Alcohol consumption inhibits locomotor activity [32]. In our hand, locomotor activity started to decrease in alcohol-drinking rats after 7 d. Rearing activity was also decreased. Notably, the distances travelled and rearing were not different compared to control rats at the end of the 28-day alcohol exposure, suggesting adaptation and compensation. Discontinuation of alcohol intake results in the nervous system hyperactivity and dysfunction [33]. EWS is the most important evidence indicating the presence of physical alcohol dependence either in humans or in experimental animals [34]. Withdrawal symptoms include increases in stereotype behaviors, tail stiffness, hyper-reflexia, agitaion, and anxiety [35]. In our study, rats exhibited obvious withdrawal signs after alcohol discontinuation. In line with previous studies [36], [37], the global score of EWS was highest at 6 h. Our results indicated that 28-d of continuous 6% alcohol drinking induces physical dependence in rats.

Activated ERK phosphorylates cellular targets or translocates into the nucleus where it activates specific gene transcription factors [38]–[40]. By regulating cellular activities and gene transcription, the ERK cascade transduces the activity of a variety of extracellular and intracellular signals into enduring changes in the central nervous system. Several studies have shown that alcohol exposure alters ERK phosphorylation. Moderate doses of acute alcohol (1.5–3.5 g/kg) produce a dose- and time-dependent decrease in phosphorylated ERK in mouse cortex [41]. Another study extended these findings by showing that acute alcohol can reduce phosphorylated ERK in the cerebral cortex and Hip in both young and adult rats [42]. Forced exposure to chronic ethanol vapor suppressed ERK phosphorylation in the Amy, cortex, cerebellum and CPu in rats [43]. Our results showed that chronic or acute exposure to alcohol significantly decreased ERK phosphorylation in the NAc, CPu, Amy, Hip, and PFC. The most intriguing finding of the present study is that ERK phosphorylation also decreased significantly in the NAc, which is inconsistent with the finding of Sanna *et al*. wherein they found only minor and mostly non-significant changes [43]. This might be the result of the different sample sizes (n = 5 *v.s.* n = 10) used or the duration of the induction periods (12 days *v.s.* 28 days). Although drugs of abuse possess diverse neuropharmacological profiles, activation of the mesocorticolimbic system, particularly the NAc, Amy, CPu, PFC and Hip via dopaminergic and glutamatergic pathways, constitutes a common pathway by which various drugs of abuse mediate their reinforcing effects [12]. The similar alterations of ERK have been thought to contribute to the drug’s rewarding effects and to the long-term maladaptation induced by drug abuse (including cocaine, amphetamine, Δ^9^-tetrahydrocannabinol, nicotine, morphine and alcohol) [44]. Although long-term alcohol exposure induced significant inhibition of ERK phosphorylation, distinct patterns of p-ERK/ERK ratio were observed in different brain regions. It may reveal that these different brain regions play different roles and have different sensitivity to alcohol. Together, our results demonstrated that chronic or acute alcohol exposure induces decreased ERK phosphorylation in the brain reward circuit. Alcohol inhibits glutamatergic neurotransmission, primarily by acting on ionotropic glutamate receptors (iGluRs). Many reports have demonstrated that acute ethanol exposure inhibits NMDAR channel function in the Hip, cerebellum, cerebral cortex, NAc, Amy and VTA [45]–[47]. After chronic alcohol exposure, the number of NMDA receptor complexes was increased [48]–[50], perhaps indicating an adaption to the chronic presence of ethanol to maintain receptor activity. These results confirmed that the activity of glutamate receptors but not the number of these receptors is inhibited by chronic alcohol exposure. One well-characterized cascade includes the calcium/calmodulin-dependent inhibition of adenylyl cyclase and inhibition of cAMP-dependent protein kinase (PKA). PKA then triggers the inhibition of ERK signaling cascade, resulting in decreased ERK phosphorylation and nuclear translocation and further down-regulation of gene transcription [51], [52]. Our recent study has demonstrated that continuous alcohol drinking inhibits the phosphorylation of ERK, and this inhibition is correlated with a decrease in the phosphorylation of CaMKII (Thr286) in hippocampal CA1 and DG subregions [53].

The GluN1 subunit forms the molecular backbone of functional NMDA receptors and has been hypothesized to play a role in chronic alcohol consumption. To address this issue, we examined the expression and phosphorylation levels of the GluN1 subunit. Long-term alcohol consumption induced significant up-regulation of GluN1 subunits in all five brain regions. Enhanced GluN1 subunit mRNA expression was evident in samples from chronic ethanol-exposed animals in amygdala neurons [54]. Previous studies have shown that alcohol consumption is accompanied by increased GluN1 protein levels in the Hip [55], Amy [48], striatum and medial PFC [56]. In Kroener *et al.* study, they observed that chronic alcohol exposure lead to an increase in expression of GluN1 subunit in the insoluble postsynaptic density fraction, but this increase was more transient and was no longer observed after 1 week of withdrawal [57]. The up-regulation of GluN1 subunits caused by chronic alcohol consumption may be a compensatory reaction that leads to recovery of NMDAR functional activity following ethanol-induced GluN1 inhibition [58], [59].

Protein phosphorylation has been recognized as a major mechanism for the regulation of NMDA receptor function [60], [61]. In our hand, the ratio of phospho/total-GluN1 was significantly lower in all brain regions examined. Phosphorylation of GluN1 at Ser897 alters NMDA receptor activity by regulating its sensitivity to glutamate or by activating downstream signal transduction pathways [62]. A serine at position 897 in the C1 cassette represents the major PKA phosphorylation site of the GluN1 subunit [63]. Dopamine D1 receptors have been shown to directly bind GluN1 and GluN2A subunits [64], [65], these results suggest that the phosphorylation status of the GluN1 S897 site may be a critical factor that regulates the overall sensitivity of NMDA receptors to alcohol. However, a previous study showed that conditions that favor the phosphorylation of S897 in the GluN1 subunit had no effect on the acute alcohol sensitivity of NMDARs [66]. Aside from methodological differences between Xu *et al.* and the present study, it is also possible that neuronal NMDA receptors exist in a complex intracellular environment characterized by a wide array of signaling proteins that are presumably missing in a heterologous cell expression system like human embryonic kidney cells. Our results confirmed previous observations that continuous alcohol drinking greatly increases GluN1 in both total protein and its phosphorylated version [67]. Moreover, our results also showed the ratio of phospho/total-GluN1 subunit was reduced in these brain regions studied, which indicate that total-GluN1 were increased greater than phospho-GluN1 in the continuous alcohol drinking group. it is plausible that chronic alcohol use, by long-term inhibition of NMDA function, triggers compensatory adaptations. Zhao *et al.* have demonstrated that decreased phospho/total-GluN1 is consistent with decreased NMDAR function [68]. Moreover, we also demonstrated that acute exposure to alcohol induced increases in GluN1 phosphorylation in rat brain. The upregulated phosphorylation of GluN1 may lead to recovery of NMDAR functional activity under the acute effect of alcohol [30]. These effects of alcohol on the GluN1 may underlie the mechanisms that compensate for alcohol-induced inhibition of NMDARs. Previous study documented a profound increase in phospho-ERK in response to pharmacological activation of NMDA receptors in hippocampal, cortical and striatal neurons [39]. Administration of the NMDARs antagonist MK-801 prevented the increase in ERK phosphorylation in the Amy in alcohol withdrawn rats [69]. Thus, it may be speculated that the decreased ERK phosphorylation is associated with the inhibition of phospho-GluN1 by long-term alcohol drinking.

In conclusion, the findings of the present study indicate that alcohol can affects phosphorylation of ERK and GluN1 in brain reward circuits. JNK and p38 phosphorylation in mesocorticolimbic areas were not significantly influenced by this treatment. Our findings support previously reported associations between GluN1-ERK and dependence to alcohol and other substances, and emphasize the relevance of decreased phosphorylation of GluN1 and ERK in the mesocorticolimbic system for chronic alcohol exposure.

## Supporting Information

Figure S1 **Schematic representation showing the approximate location of the brain regions excised and used for analysis.** NAc: nucleus accumbens; CPu: caudate putamen; Amy: amygdala; Hip: hippocampus; PFC: prefrontal cortex (from Paxinos & Watson, 2005).
(TIF)

Table S1 **Rating scale for some behavior signs induced by ethanol withdrawal in rats.**
(DOCX)
