# Supplementary figures and images for: Differential Phosphorylation of GluN1-MAPKs in Rat Brain Reward Circuits following Long-Term Alcohol Exposure
Source: PLoS One. 2013 Jan 23;8(1):e54930. doi: 10.1371/journal.pone.0054930 (PMC3553008; doi:10.1371/journal.pone.0054930)

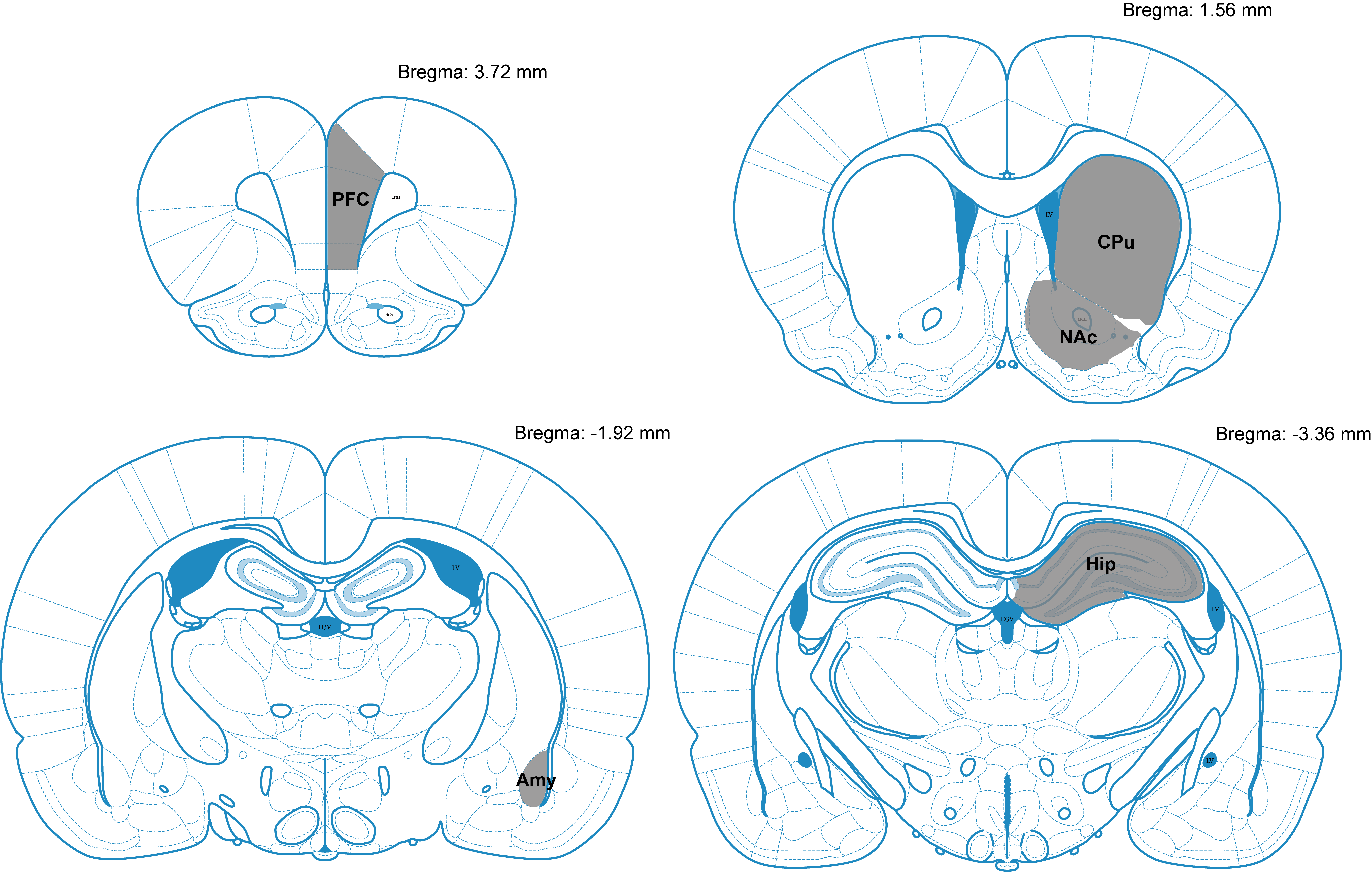

Supplement: Figure S1 — Schematic representation showing the approximate location of the brain regions excised and used for analysis. NAc: nucleus accumbens; CPu: caudate putamen; Amy: amygdala; Hip: hippocampus; PFC: prefrontal cortex (from Paxinos & Watson, 2005). (TIF) [file pone.0054930.s001.tif]
