# Supplementary material for: Differential Phosphorylation of GluN1-MAPKs in Rat Brain Reward Circuits following Long-Term Alcohol Exposure
Source: PLoS One. 2013 Jan 23;8(1):e54930. doi: 10.1371/journal.pone.0054930 (PMC3553008; doi:10.1371/journal.pone.0054930)
Supplement: Table S1 — Rating scale for some behavior signs induced by ethanol withdrawal in rats. (DOCX) [file pone.0054930.s002.docx]

**Table S1.** Rating scale for some behavior signs induced by ethanol withdrawal in rats

| **Signs** | **Scoring** |
| --- | --- |
| Stereotyped behaviors* | 1: rats showing only one stereotyped behavior |
|  | 2: two stereotyped behavior |
|  | 3: three stereotyped behavior |
|  | 4: four stereotyped behavior |
|  | 5: all of stereotyped behavior |
| Agitation | 1: rats showing mild or moderate irritability |
|  | 2: very irritable |
|  | 3: handling vocalization and moderately aggressive |
|  | 4: handling vocalization and very aggressive |
|  | 5: spontaneous vocalization and very aggressive |
| Tail stiffness | 1: mild tail rigidity |
|  | 2: moderate tail rigidity |
|  | 3: tail rigidity but mildly flexible during ambulation |
|  | 4: tail rigid and not flexible during ambulation |
|  | 5: tail very rigid and not flexible during ambulation |
| Abnormal posture | 1: mild head-down, back-hunched |
|  | 2: moderate head-down, back-hunched |
|  | 3: prominent head-down, back-hunched |
|  | 4: in addition hind legs wide apart |
|  | 5: in addition forelimbs apart |
| Abnormal gait | 1-2: mild difficulty in ambulating and rearing normal |
|  | 3-4: moderate difficulty in ambulating and rearing |
|  | 5: prominent difficulty in ambulating and no rearing |

*Grooming, sniffing, head weaving, gnawing and chewing.
